# Bibliographical

**Published:** 1895-05

**Authors:** 


### Bibliographical.

Catching's Compendium of Practical Dentistry for 1894—B. H. Catching, Editor and Publisher: Atlanta, Georgia.

This compend or digest of periodical literature of the Dental profession is just at hand, and in looking over it with some care, we do not hesitate to say that it is an improvement on a former volume of the same work. Dr. C. is evidently becoming more perfect by practice. Either that, or more time and care has been exercised in selecting and presenting the extracts. It is a work specially valuable to the busy dentist, and indeed valuable to every one. The student can consult it with much profit.

The work is divided into seven departments, namely: Operative Dentistry, Prosthetic Dentistry, Crown and Bridge-Work, Orthodontia, Dental Medicine, Oral Surgery and Miscellaneous. A very full index is given in each of these departments, so at a glance one can readily find any special subject or item.

This work has each year become more and more important and desirable for the dentist, so that now a dentist's library is not complete without this work. It can be obtained of the author, and through any dental depot, and doubtless in any book store.

